# Zolpidem in Progressive Supranuclear Palsy

**DOI:** 10.1155/2013/250865

**Published:** 2013-05-09

**Authors:** Sandip K. Dash

**Affiliations:** Department of Neurology, Apollo Hospitals Dhaka, Basundhara R/A, Dhaka 1229, Bangladesh

## Abstract

Progressive supranuclear palsy (PSP) is a progressive neurodegenerative disorder, characterized by motor symptoms, postural instability, personality changes, and cognitive impairment. There is no effective treatment for this disorder. Reduced neurotransmission of GABA in the striatum and globus pallidus may contribute to the symptoms of motor and cognitive symptoms seen in PSP. Zolpidem is a GABA agonist of the benzodiazepine subreceptor BZ1. Here a nondiabetic, normotensive case of PSP is (Progressive Supranuclear Palsy) described, which showed improvement in swallowing, speech, and gaze paresis after zolpidem therapy and possible mechanism of actions are discussed. However, more trials are needed with large number of patients to confirm the effectiveness of zolpidem in progressive supranuclear palsy.

## 1. Introduction

Progressive supranuclear palsy (PSP) is an atypical parkinsonian syndrome characterized by motor symptoms, postural instability, personality changes, and cognitive impairment. It is refractory to treatment. 

The histopathology is prominent in basal ganglia, subthalamic nucleus, brainstem nuclei, dentate nucleus of cerebellum, and frontal cortex. Microglial activation is an useful indicator of neuronal injury reflecting disease severity. Activated microglia express peripheral benzodiazepine binding site, thought to be a mitochondrial protein unrelated to the GABA (Gamma-aminobutyric acid) A receptor complex, the site of central benzodiazepine binding site. PET (positron emission tomography) study is also in concordance with the known neuropathological distribution of microglial activation in basal ganglia, brainstem, cerebellum, and frontal lobe, seen in this disorder [1]. Reduced neurotransmission of GABA in the striatum and globus pallidus could contribute to the symptoms of motor and cognitive symptoms seen in PSP [2] drugs that act in the gabaergic system in the basal ganglia may be helpful in this disorder. The destruction of the basal ganglia output systems may explain the lack of responsiveness to L-dopa therapy of PSP patients. 

Zolpidem is a GABA agonist of the benzodiazepine subreceptor BZ1. The highest density is found in the output structures of basal ganglia. A double-blind placebo controlled study of 10 patients having PSP had improvement in motor function and saccadic eye movements with zolpidem compared to placebo [3]. However, in a case report [4], zolpidem produced improvements in vertical gaze palsy and Parkinsonism, but the effect lasted for 4 weeks only and was not repeatable 2 months later. In another case report [5] it was seen that zolpidem CR (controlled release) caused a delay in improvement in motor and ocular movements in a patient of PSP, and the improvement lasted over 6 months. The authors opined that a threshold level may be required before symptomatic improvement is noted or there may be alteration in benzodiazepine receptor BZ1 following prolonged and repetitive stimulation by zolpidem CR. 

## 2. Case Report

8-year nondiabetic, normotensive male with akinetic rigid syndrome for last 3 years on levodopa was admitted for irritability, indistinct speech, vertical gaze paresis, and unable to take food by mouth and insomnia. His son revealed that he was having frequent falls while walking since 3 years, insomnia, and decreased memory. He was found to be aggressive, interfering with interaction, irritable, agitated with poor attention, dysphagia requiring tube feeding, with a poorly comprehensible sounds, vertical gaze paresis, symmetric rigidity, proximal more than distal, retrocollis, unable to walk even with walker without any pyramidal tract signs or bladder involvement. His behavioral symptoms and poor attention prevented detailed neuropsychological assessment. The patient was diagnosed to have probable PSP as per NINDS criteria. Routine blood tests, serum homocystein, blood sugar were normal, and MRI Brain was unremarkable. The levodopa dosage was increased but having no effect, and the patient was treated with donepezil, quetapine, and on nasogastric feeding. Subsequently, levodopa dosage was reduced as it had no effect. Tab zolpidem, 5 mg, was given for insomnia at night. Interestingly, on second day, 2 hours after zolpidem, the son noticed that the patient's speech was better than before. Basing on the various case reports, zolpidem CR, 12.5 mg, was given twice daily instead of zolpidem with the informed consent of the patient's son. After 15 days of giving zolpidem CR, the patient's speech improved with most of the words comprehensible, and the patient was able to swallow semisolid food by mouth. Subsequently, dysphagia improved with occasional cough to swallow fluid, and nasogastric tube was subsequently removed. His speech which was indistinct became better and understandable and there was an improvement in vertical gaze, aggressiveness, and agitation but no improvement in mobility and rigidity. After 2 months, zolpidem CR was discontinued. The patient is still able to take food by mouth, speech is clear, and gaze paresis improved 4months after discontinuation of zolpidem CR. The patient is still on followup, and it is to be seen how long this improvement is persisting. 

## 3. Discussion

It is reported that zolpidem CR produced a sustained improvement in facial and vocal expression, oropharyngeal coordination, gaze paresis, and motor skills. The authors opined that this may be due to different pharmacodynamic properties in comparison to immediate release preparation of zolpidem [5]. 

In our case 2, weeks delay between the start of treatment and improvement may be due to the time taken to GABAergic stimulation time to alter the GABAergic output pathway, and a threshold level of output pathway may be required for having a symptomatic improvement. Another possibility may be that prolonged stimulation may be required to cause alterations in benzodiazepine subreceptor BZI. The improvement in gaze may be due to inhibition of substantia nigra zolpidem that could also activate superior colliculus [2]. The duration of the disease may be a key factor determining the sustained effect. This may be due to the severity of neuronal loss in cortical and subcortical structures as the duration of PSP prolongs. 

The only side effect observed during zolpidem CR treatment was drowsiness which passed away after a week in spite of continued treatment. No other adverse events were encountered. 

## 4. Conclusion

It requires more trials involving large number patients with varying duration of PSP to find out the effect of Zolpidem in swallowing and oculopharyngeal movements. If it will be found to be effective, then it will be a therapeutic option in this disease where no effective treatment is available at present. 
